# Advancing Prostate Cancer Survivorship through Transatlantic Consortium Science: The iCCaRE Initiative

**DOI:** 10.1158/2767-9764.CRC-26-0165

**Published:** 2026-09-03

**Authors:** Folakemi T. Odedina, Roxana Dronca, Kimlin Tam Ashing, Ernest Kaninjing, Solomon Rotimi, Che Ngufor, Arnold Merriweather, Vinessa Gordon, Emelina Asto-Flores, for the iCCaRE Consortium Group, Kayode Adeniji, Emmanuel Agboola, Uju Aliche, Usman M. Aliyu, Kimlin Tam Ashing, Gladys Asiedu, Emelina Asto-Flores, Paul Badejo, Kim Barbel Johnson, Patrick Beckford, Renaldo Blocker, Opeyemi Bolajoko, Timethia Bonner, Ivey Breland, Amanda Caballero, Rickey Carter, Alex Cattnell, Ewan Cobran, Gerardo Colon-Otero, John A. Copland, Getachew Dagne, Opeyemi C. De Campos, Melissa Doty, Roxana Dronca, Ebenezer Erefah, Kaila Falzon, Damian Francis, Michelle Fudge, Dottington Fullwood, Jeffery Gainer, Dee Glaser-Boivin, Mahesh Gokara, Vinessa Gordon, Antony Hill, Rakenya Hinsom, Emeka J. Iweala, Ernie Kaninjing, Adam Kase, Adam Kase, Keith Knutsen, Daniel Lee, Sandra Casanova Leyva, Carolyn R. Lindeman, Collisa Mahin, Michael Maniaci, Trevanne Matthews Hew, Trevanne R. Matthews Hew, John McCall, Jada K. Melton, Arnold J. Merriweather, Luisa Mitchell, Cassandra Moore, Che Ngufor, Lucia Nolan, Abimbola Odedina, Folakemi Odedina, Catherine Oladoyinbo, Esther Olasehinde, Abimbola F. Onyia, Ademoola Popoola, Jordan P. Reynolds, Solomon O. Rotimi, Tracie Scales, Hanna Sledge, Jade Thompson-Reeves, Oluwaseyi Toye, Claudia Valles Duarte, Kaitlin Van Voorhis, Charles F. Waldon, Kim Walsh-Childers, Christopher Williams, Floyd Willis, Floyd Willis, Quincy Wimberly, Ayinde Yahaya, Clayton Yates, Kathleen Yost, Mary Ellen Young, Tingyu Zou

**Affiliations:** 1 Division of Hematology/Oncology, Mayo Clinic, Jacksonville, Florida.; 2 Inclusive Cancer Care Research Equity (iCCaRE) Consortium, Global Consortium, Jacksonville, Florida.; 3 Prostate Cancer Transatlantic Consortium (CaPTC), Global Consortium, Jacksonville, Florida.; 4 City of Hope Comprehensive Cancer Center, Beckman Research Institute, Duarte, California.; 5 School of Health and Human Performance, Georgia College & State University, Milledgeville, Georgia.; 6 Covenant University, Ota, Nigeria.; 7 Robert D. and Patricia E. Kern Center for the Science of Health Care Delivery, Mayo Clinic, Rochester, Minnesota.

## Abstract

Black/African American populations, globally, bear the greatest prostate cancer burden compared with other racial/ethnic groups. Addressing prostate cancer disparities in Black men is complex, requiring a patient-centered, community-centric, and comprehensive approach. This study integrates Inclusive Cancer Care Research Equity (iCCaRE) Consortium findings and broader literature to address unmet needs in survivorship care. The iCCaRE Consortium implemented five complementary pilot studies using mixed-methods designs, including feasibility trials, qualitative research, and observational analyses across the United States and sub-Saharan Africa. Descriptive statistics, subgroup analyses, and qualitative thematic analyses were performed across these studies. Collectively, projects addressed multilevel determinants, including social, behavioral, and biological drivers of disparities. Project 1 implemented a Virtual Realty Assistant across clinical and community settings, with ongoing evaluation in pragmatic trials. Project 2 showed strong patient preference for home-based cancer care. Project 3 demonstrated trends toward improved health-related quality of life. Project 4 identified key themes influencing survivorship experiences. Project 5 identified biological mechanisms linking stress, immune modulation, and symptom burden. The iCCaRE pilot projects demonstrate the feasibility and early impact of a consortium-based, transdisciplinary approach to addressing prostate cancer disparities in Black men. These findings highlight the importance of integrating patient-centered interventions, community engagement, and biological insights to inform scalable strategies aimed at improving survivorship outcomes and advancing health equity. The iCCaRE Consortium demonstrates that a transatlantic, patient-centered, and multidisciplinary approach integrating social, clinical, and biological factors can generate scalable strategies to improve survivorship outcomes and advance equity in prostate cancer care for Black men.

**Significance:**

Black men experience the highest global burden of prostate cancer, yet disparities persist due to fragmented research and limited infrastructure. The iCCaRE Consortium demonstrates how coordinated, community-engaged consortium science can accelerate equity-driven research, expand minority investigator leadership, and generate sustained funding and scholarly productivity. By integrating survivors, advocates, scientists, and clinicians across continents, iCCaRE establishes a scalable model to reduce prostate cancer disparities and advance health equity worldwide.

## Introduction

Black/African American populations, within and outside the United States, bear the greatest prostate cancer burden compared with other racial/ethnic groups. In the United States, Black men have a 1 in 6 lifetime probability of developing prostate cancer and a 1 in 35 lifetime probability of dying from prostate cancer (1). Prostate cancer mortality in Black populations is more than twice that observed in White men, representing the highest mortality burden among all racial and ethnic groups. The prostate cancer disparities experienced by Black men in the United States are a microcosm of the global burden of prostate cancer in Black men (2, 3). For example, the World Health Organization estimated that 9 of the top 10 countries leading in prostate cancer mortality in the world are all Black nations (4, 5). Existing literature demonstrates persistent disparities in prostate cancer across the continuum of care, including later stage at diagnosis, differences in treatment access and quality, and poorer survivorship outcomes among Black men compared with other populations (6–9). Prior studies from Inclusive Cancer Care Research Equity (iCCaRE) Consortium members have also identified critical gaps at the point of diagnosis, including limited psychosocial support, health literacy challenges, and barriers related to social determinants of health (SDOH; refs. 10, 11). This study builds upon these findings using a consortium science approach to address disparities through coordinated, transdisciplinary collaboration.

Addressing prostate cancer disparities in Black men requires a patient-centered, community-centric, and comprehensive approach, with prostate cancer survivors, advocates, scientists, and clinicians working closely together to conceptualize, conduct, disseminate, and translate research into clinical and community applications. Based on this principle, the appropriate approach to use in addressing prostate cancer disparities in Black men globally is the transdisciplinary, multisectoral, stakeholder-partnered, and coordinated consortium science (12, 13).

### Consortia rationale and focus

According to the National Cancer Institute’s Epidemiology and Genomics Research Program, a consortium is “a group of scientists from multiple institutions who have agreed to participate in cooperative research efforts involving activities such as methods development and validation, pooling of information from more than one study for the purpose of combined analyses, and collaborative projects.” Common characteristics of consortia are that the group works and thinks together, collaborators are equal partners, there is shared leadership, and collaborators trust each other. The need for establishing a consortium arose due to the complexity of health disparities and the need for a unique approach to better understand and address complex chronic diseases, such as cancer. Consortium science continues to gain ground because health disparities research cannot be solved individually, and there is a critical need for shared resources and expertise to make the “whole bigger than the sum of its parts.” With consortia, researchers are able to address scientific questions that cannot be addressed individually due to scope, skills, resources, population size, expertise, scientific knowledge, and technologies. This has ultimately led to research productivity and high-quality research.

The significance of consortium science is underscored by federal funding opportunities focused on leveraging consortia to address health disparities. In 2021, the Department of Defense Congressionally Directed Medical Research Programs Prostate Cancer Research Program released the Health Equity Research and Outcomes Improvement Consortium Award phase I to support a synergistic, multi-institutional collaboration incorporating innovative and translational approaches that have the potential to make a major impact on (i) improving quality of life to enhance outcomes and overall health and wellness for those affected by prostate cancer and (ii) advancing health equity and reducing disparities in prostate cancer. The iCCaRE Consortium was one of the two consortia funded for this award. The primary goal of this article is to report the impact of consortium science on prostate cancer disparity research scholarship, focusing on the achievements of the five research projects.

### iCCaRE Consortium overview

**Figure 1.**
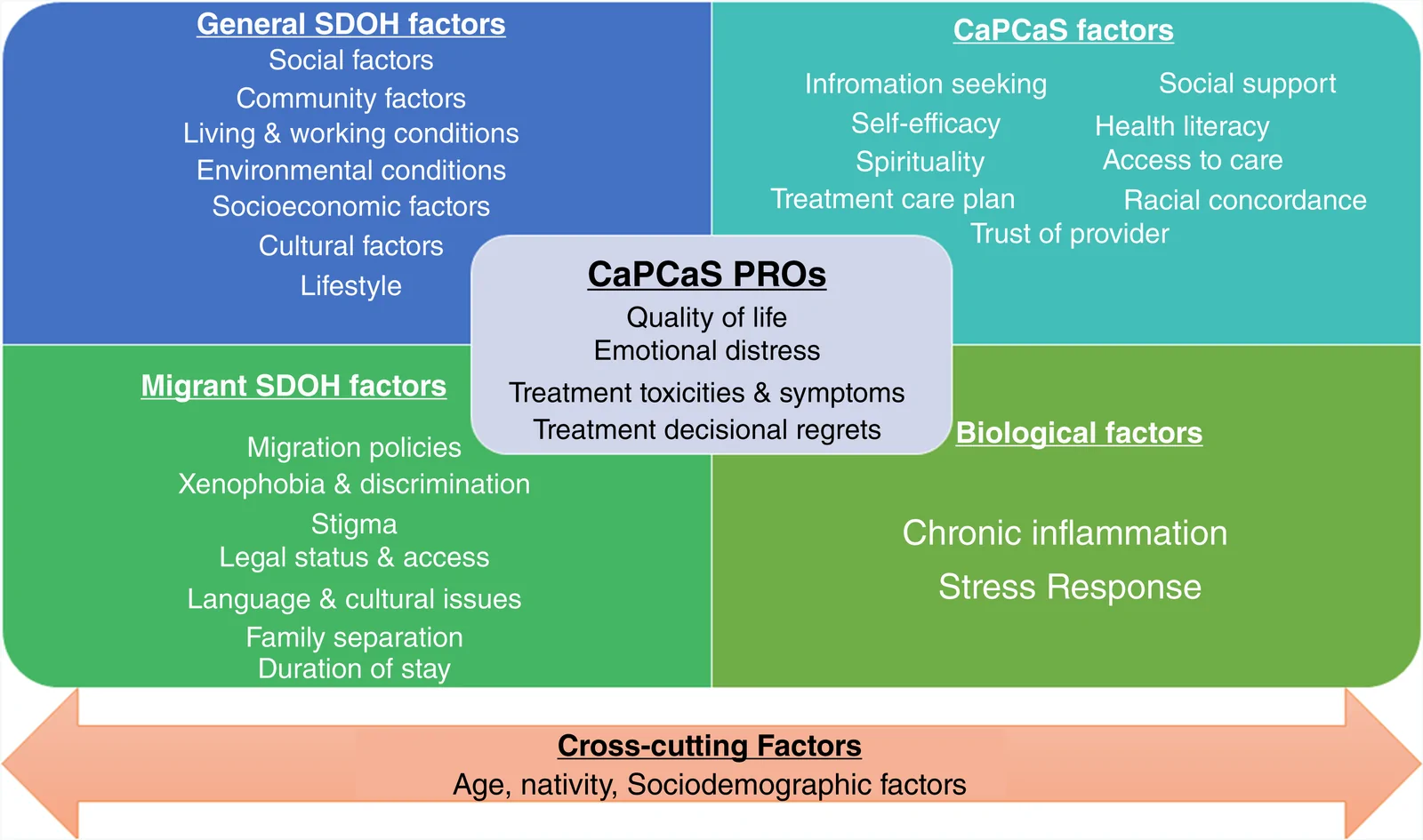
iCCaRE Consortium CaPCaS PROs model for Black men. Conceptual framework illustrates the multilevel determinants of CaPCaS PROs among Black men. The model integrates biological factors (e.g., inflammation, stress response), general and migrant-specific SDOH, and CaPCaS-specific factors (e.g., health literacy, social support, access to care, and trust in providers) to influence key outcomes, including quality of life, emotional distress, treatment toxicities, and decisional regret. Cross-cutting sociodemographic factors such as age, nativity, and socioeconomic status are also depicted.

The iCCaRE Consortium focuses on achieving health equity by reducing disparities associated with prostate cancer diagnosis, treatment, and survivorship and improving patient-reported outcomes (PRO) related to prostate cancer. Based on the science of survivorship (SOS), the consortium takes a unique approach to study and address prostate cancer disparities through within-group differences among US-born Black men, African immigrant men in the United States, and indigenous West African men to tailor the research and interventions developed for these subpopulations. The guiding model for the consortium is the iCCaRE Consortium prostate cancer care and survivorship (CaPCaS) PRO model for Black men (Fig. 1), which is based on over 25 years of prostate cancer research by members of our consortium (10, 11, 14–34). The central hypothesis of the iCCaRE Consortium is that providing navigation to address SDOH factors, improving CaPCaS-related factors, and mediating biological factors will lead to an improvement in PROs. The overall goal of the consortium is to optimize prostate cancer diagnosis experiences, treatment, and survivorship by developing feasible, innovative, and sustainable interventions that will reduce prostate cancer disparities experienced by men of African ancestry. In line with this goal, the objectives for the iCCaRE Consortium are as follows:

1. Establish new global scientific collaborations, expand research infrastructure/resources, and expand existing patient cohorts.
2. Plan, develop, and pilot test tailored, technology-infused interventions using artificial intelligence (AI) focused on SDOH and CaPCaS factors to improve PROs of Black patients.
3. Explore modifiable sociobiological factors affecting the PROs of ethnically diverse Black men.

Five institutions, three community-based organizations (CBO) and two consortia collaborated to form the iCCaRE Consortium. The academic institutions are Mayo Clinic, City of Hope, Georgia College & State University, Florida A&M University, and Covenant University in Nigeria. The CBOs are the American Legion Post 197, Black Male Prostate Cancer Coalition (BMPCC), and Project Pink Blue. The iCCaRE Consortium significantly leveraged the Prostate Cancer Transatlantic Consortia (CaPTC) infrastructure, resources, and expertise. Additionally, CaPTC partnered with the African-Caribbean Cancer Consortium to build the iCCaRE Consortium. In this article, we discuss the impact of the iCCaRE Consortium on research scholarship.

#### Administrative and scientific management of iCCaRE Consortium

**Figure 2.**
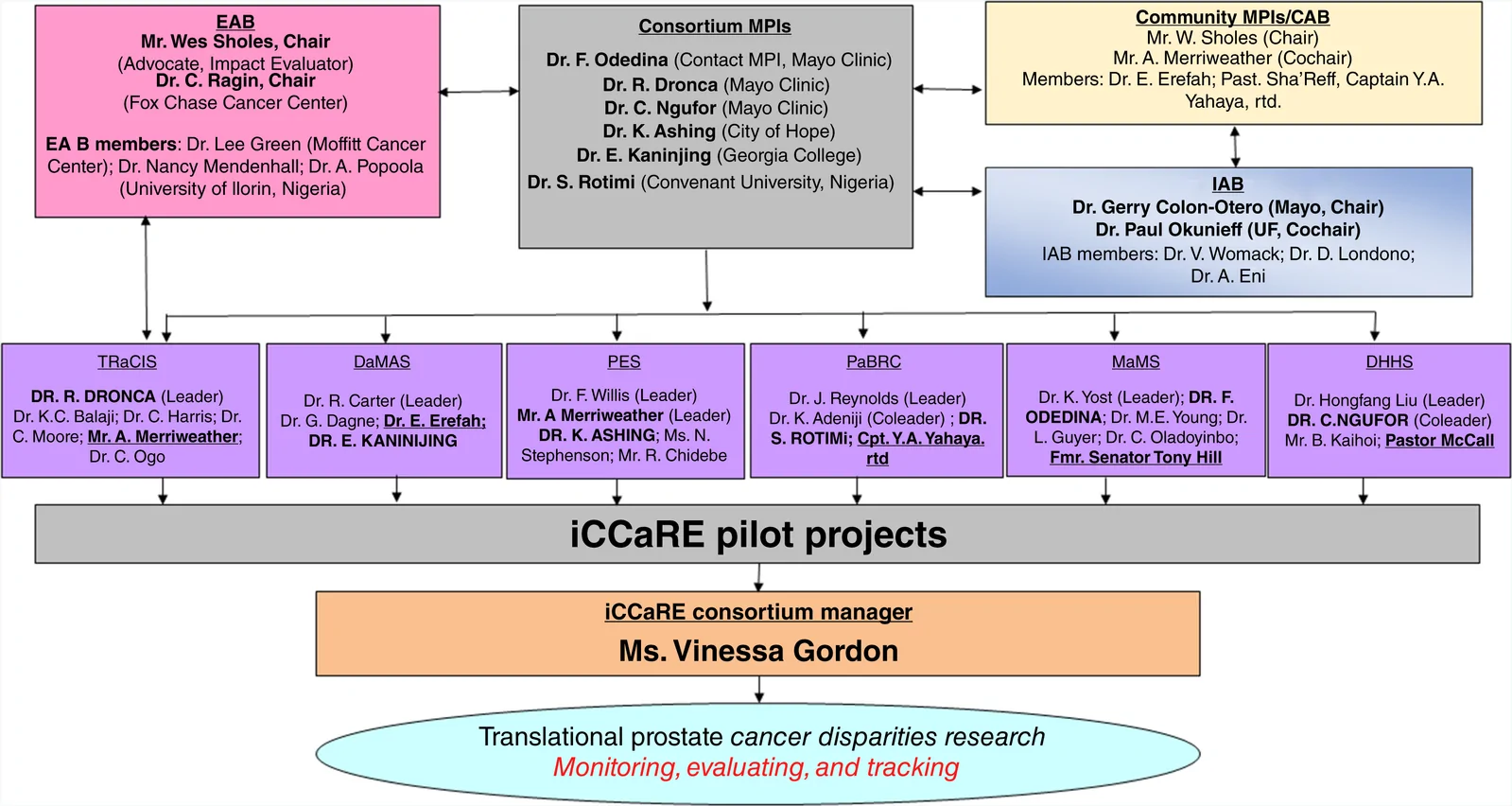
iCCaRE Consortium administrative leadership structure. Organizational structure of the iCCaRE Consortium highlighting governance, leadership, and collaborative components. The model includes Consortium multiple PIs (MPI), internal and external advisory boards, core service units [e.g., Translational Research and Clinical Intervention Service (TRaCIS), Pathology and Biospecimen Resource Core (PaBRC), DaMAS], and the CAB. The structure emphasizes multidisciplinary coordination, community engagement, and integration of scientific and operational functions to support consortium activities. MPI/AC liaisons to each core are indicated in all uppercase, and prostate cancer survivors/community scientists are shown underlined. EAB, external advisory board; IAB, internal advisory board.

Objective 1 was to establish new global scientific collaborations, expand research infrastructure/resources, and expand existing patient cohorts to advance the mission and community engagement research priorities of the iCCaRE Consortium. We leveraged the resources and infrastructure of iCCaRE member institutions/organizations/consortia, as well as the expertise of collaborating investigators (including consumer advocates), to establish cores/services that supported the five iCCaRE projects. Each core/service of iCCaRE is specialized unit or resource that provides essential support, infrastructure, or expertise to advance the consortium’s scientific and operational goals (Fig. 2). The Administrative Core (AC), with strong support from Advisory Boards, fulfilled the administrative and scientific functions for the Center. The Consortium multiple principal investigators (PI) were responsible for each project’s overall scientific design, provided oversight and coordination of all activities, oversaw all fiscal aspects of the Consortium, and ensured the highest level of scientific rigor and quality.

#### iCCaRE research projects

**Figure 3.**
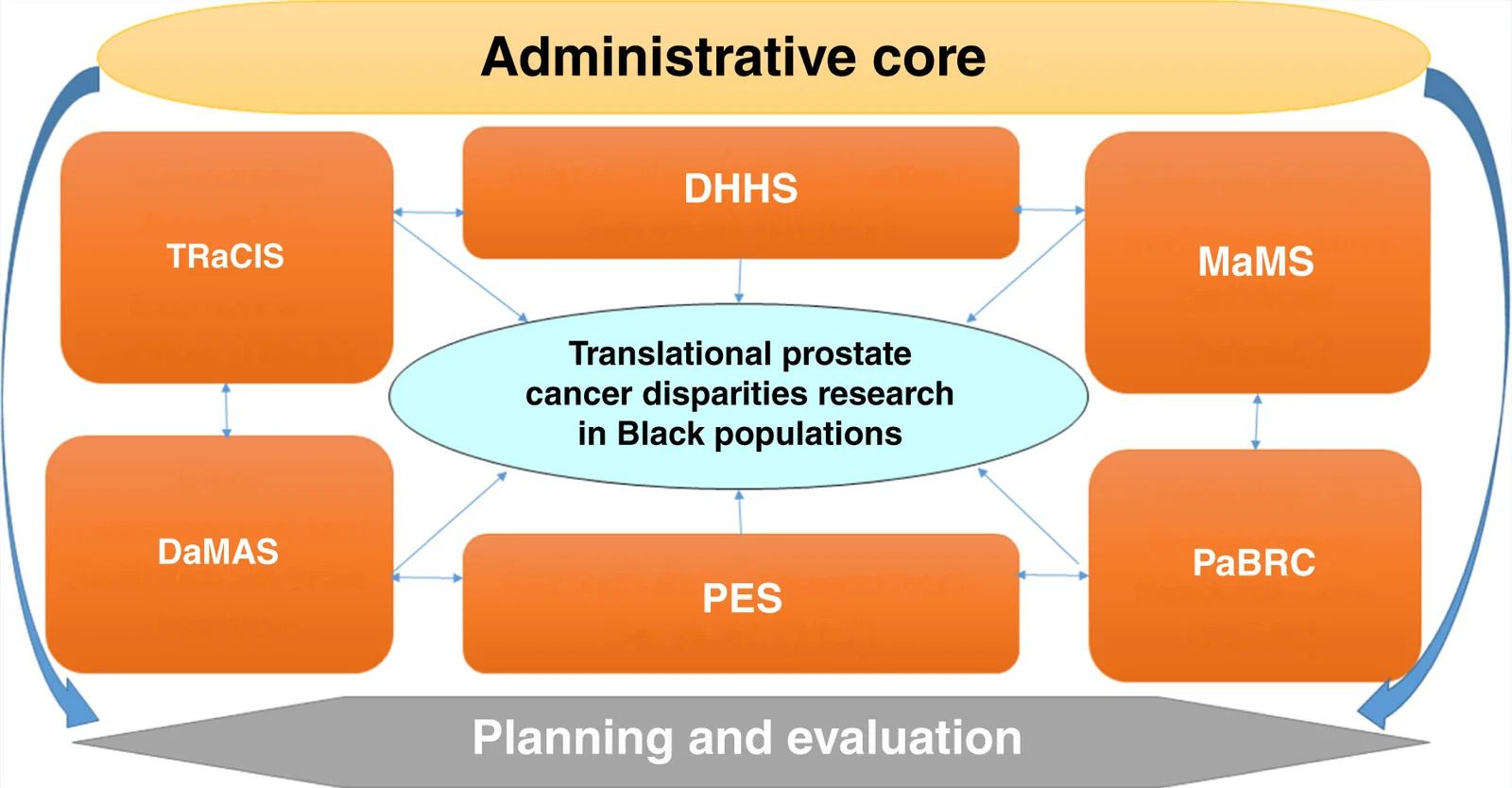
iCCaRE Consortium cores and shared services. Overview of the core infrastructure supporting the iCCaRE Consortium. Core components include AC, MaMS, DaMAS, Pathology and Biospecimen Resource Core (PaBRC), Translational Research and Clinical Intervention Services (TRaCIS), Partnership Engagement Services (PES), and Digital Health and Human Services (DHHS). These cores provide integrated support for translational research, data management, community engagement, and evaluation across all consortium projects.

Led by a scientific PI and a community PI (who is a prostate cancer survivor), five projects were implemented over 2 years, between 2022 and 2024. The iCCaRE projects were based on the CaPCaS PROs model and facilitated by the consortium cores/services (Fig. 3). Table 1 provides a summary of the projects and team members.

**Table 1.** iCCaRE Consortium pilot projects.

Title	Study populations	Scientific MPIs	Community MPI
Specific aim achieved through pilot projects 1–3: Plan, develop, and pilot test tailored, technology-infused interventions using AI focused on SDOH and CaPCaS factors to improve PROs of Black patients.			
Project 1: Feasibility of a Point of Prostate Cancer Diagnosis Intervention for Newly Diagnosed Black Men	Black/African American, African immigrant, and Caribbean immigrant men diagnosed with prostate cancer in Florida, USA	F.T. Odedina, PhD and C. Ngufor, PhD	A. Merriweather
Project 2: Feasibility of Patient-Centered Home Care (PCHC) to Reduce Disparities in High-Risk Black Men with Advanced Prostate Cancer (CaP)	Black men with advanced or metastatic prostate cancer from the Mayo Clinic Florida oncology clinical practice who are requiring active standard anticancer therapy	R. Dronca, MD & Trevanne Matthews Hew, MBBS	Former Senator Anthony “Tony” Hill
Project 3: Whole Person Survivorship Care to Improve Health-Related Quality of Life (HRQOL) in Black Prostate Cancer Patients	Black/African American, African immigrant, and Caribbean immigrant men who are survivors of prostate cancer	K.T. Ashing, PhD	Pastor John McCall
Specific aim achieved through pilot projects 4 and 5: Explore modifiable sociobiological factors affecting the PROs of ethnically diverse Black men.			
Project 4: Grounded Theory Study of the Social Determinants of Migrant Health Factors Impacting Prostate Cancer Care and Survivorship among Sub-Saharan African Immigrant Men Diagnosed with Prostate Cancer	SSA immigrant men diagnosed with prostate cancer	E. Kaninjing, DrPH	Mr. Emmanuel Agboola
Project 5: Assessment of Biological Determinants of Psychoneurological Symptom Cluster among Ethnically Diverse Black Prostate Cancer Survivors	Indigenous SSA men	S. Rotimi, PhD	Capt. Ayinde A. Yahaya, rtd.

NOTE: Summary of the five pilot projects implemented within the iCCaRE Consortium between 2022 and 2024 to address prostate cancer disparities among Black men. Projects 1 to 3 focused on developing and pilot-testing patient-centered, technology-enabled interventions targeting SDOH, survivorship, and PROs. Projects 4 and 5 examined modifiable social, behavioral, and biological determinants influencing survivorship outcomes among ethnically diverse Black men. The table also highlights study populations, scientific leadership, and community PIs supporting each project.

Abbreviation: MPI, multiple PI.

#### Unique role of consumer advocates in the iCCaRE Consortium

The iCCaRE Consortium is an academic–community partnership, with each project led by scientific PI(s) and a community leader, and each of our consortium core/service including a consumer advocate. The community leaders make up the community advisory board (CAB), chaired by A. Merriweather. In addition, A. Merriweather codirects the consortium with F.T. Odedina. In line with the principles of community-based participatory research (CBPR; refs. 35–37), the consortium equitably involves all collaborating partners in the research process. The consortium includes African American, Caribbean, and African community leaders who were integrally involved in the conceptualization, design, and implementation of the consortium.

#### Continuous improvement facilitated by evaluation

Led by the program evaluation team, continuous evaluation and tracking of consortium activities were employed based on clear metrics toward the long-term impact of the consortium on expanding prostate cancer disparities research, as well as increasing the pool of underrepresented minority investigators focused on translational research through collaborative projects. Both formative and summative evaluations were employed for evaluative data collection to track the progress of the research projects and core activities and assess the congruence between objectives, activities, and outcomes.

## Materials and Methods

### Study design and framework

The iCCaRE Consortium implemented multisite, mixed-methods, prospective cohort, and pilot intervention studies guided by the SOS framework and the CaPCaS PROs model. The central hypothesis was that addressing SDOH, care-process factors, and biological mediators improves PROs among men of African ancestry diagnosed with prostate cancer.

The consortium included feasibility and nonrandomized intervention studies, with randomized components incorporated in ongoing trials. The activities of each project followed Enhancing the Quality and Transparency Of health Research reporting standards relevant to study type, for example, Consolidated Standards of Reporting Trials for pilot trials, Strengthening the Reporting of Observational Studies in Epidemiology for observational analyses, and Consolidated Criteria for Reporting Qualitative Research for qualitative components.

### Study setting and population

Participants were recruited across five academic institutions (Mayo Clinic, City of Hope, Georgia College & State University, Florida A&M University, and Covenant University in Nigeria) and three CBOs in the United States [American Legion Post 197 and BMPCC and sub-Saharan African (SSA) Project Pink Blue] between January 2022 and December 2024.

### Target populations

Eligible participants were adult men (≥18 years) of African ancestry diagnosed with prostate cancer and categorized into three predefined groups: (i) US-born Black men; (ii) African immigrant men living in the United States; and (iii) Indigenous African men living in SSA.

### Inclusion and exclusion criteria

All consortium projects’ participants met the following criteria: (i) histologically confirmed prostate cancer, (ii) ability to provide written informed consent, and (iii) ability to complete surveys in English or an approved translated language. Diagnosis of prostate cancer was confirmed by a urologist via electronic health records or verified clinical documentation for community-based participants. The exclusion criteria were cognitive impairment preventing survey completion, active psychiatric crisis preventing participation, and/or concurrent enrollment in conflicting interventional trials.

### Sample size determination

Based on each Consortium study, pilot studies were powered to detect moderate effect sizes. Observational analyses pooled data across projects to increase precision for subgroup comparisons.

### Recruitment procedures

Recruitment used standardized procedures across sites tailored to each study. Examples include clinic-based identification through electronic health records, community outreach through community organizations, referrals by survivors, and/or registry screening using the iCCaRE Community Research Registry. With oversight by the Methodology and Measures Services (MaMS), all recruitment scripts were standardized, and staff were trained using a centralized protocol manual. Recruitment logs were maintained by each project to document eligibility, refusal rates, and reasons for exclusion.

### Randomization and blinding

Due to the exploratory nature of the projects, participants enrolled in intervention studies were not randomized. In addition, participants and interventionists were not blinded. However, data analysts received deidentified coded datasets. Interim analyses were led and conducted by the iCCaRE Data Management and Analytic Services (DaMAS).

### Intervention description

For the three studies that focused on intervention, intervention fidelity was ensured through standardized manuals of procedures, training certification of staff, quarterly fidelity audits, and session recording and scoring using adherence checklists.

### Data collection

Data collection included surveys (online and in-person), qualitative interviews, and biospecimen collection for biological analyses. With guidance from iCCaRE Translational Research and Clinical Intervention Service, Pathology and Biospecimen Resource Core, MaMS, and/or DaMAS, data collection was tailored to each project using appropriate measures:

- *PROs*. Validated instruments included prostate cancer–specific quality of life, symptom burden, treatment satisfaction, mental health status, care access, and financial toxicity.
- *Clinical Data.* These data were extracted from medical records, including cancer stage and grade, treatment modality, comorbidities, and PSA levels.
- *SDOH*. Standardized measures were assessed, including insurance status, transportation access, food security, housing stability, and health literacy.
- *Biological Measures*. The biological project was conducted in Nigeria. Cases were defined as prostate cancer survivors, including men diagnosed with prostate cancer from the time of diagnosis through the rest of their lives. Controls were age-matched men without a diagnosis of prostate cancer and with PSA values less than 4 ng/mL. The final analysis included 56 cases and 95 controls. The plasma levels of tryptophan and kynurenine were assayed using the standard ELISA method, and the comparison between cases and controls was done using a Mann–Whitney U test, with statistical significance assessed using the corresponding *P* value. Laboratory staff were blinded to participant group.

The standard manuals of procedures are available as supplemental documents upon request.

### Data management

Data management was handled by DaMAS using a centralized Research Electronic Data Capture database for appropriate projects. Management of data included range checks, required field validation, audit trails, and double-entry verification for critical variables. Missing data procedures included real-time follow-up for incomplete surveys, multiple imputation for analyses when missingness >5%, and sensitivity analyses comparing complete-case results.

### Statistical analysis

DaMAS also handled the statistical analysis. Analyses were prespecified in a statistical analysis plan prior to database lock. Examples of primary analyses were mixed-effects linear regression comparing changes in PRO scores between groups over time, and adjustment for site, ancestry subgroup, age, and baseline disease severity. Examples of secondary analyses were subgroup comparisons among ancestry groups and dose-response analyses for intervention engagement. All tests were two-sided with alpha = 0.05. Effect sizes and confidence intervals were reported.

### Qualitative methods

MaMS provided oversight for all qualitative methods within the iCCaRE Consortium. For relevant studies, semistructured interviews were conducted with a purposive sample of participants, navigators, and clinicians. Interviews were recorded, transcribed verbatim, and coded independently by two analysts using a codebook developed iteratively. Discrepancies were resolved by consensus.

### Program evaluation

Under the leadership of the iCCaRE Consortium Administrative Core (AC), formative and summative evaluations were employed to assess implementation outcomes, including reach, adoption, fidelity, acceptability, and sustainability. Evaluation activities were guided by implementation science frameworks and included assessment of reach, adoption, fidelity, acceptability, and sustainability across all projects. Data sources included standardized project reports, participant-level metrics, and quarterly reviews conducted by the evaluation committee. These metrics were used to monitor implementation progress, ensure protocol adherence, and inform continuous improvement across consortium activities. Metrics were collected quarterly and reviewed by the evaluation committee.

### Community engagement and governance

Led by the Partnership Engagement Services, the study followed CBPR principles. Community representatives participated in protocol development, recruitment strategy design, interpretation of findings, and dissemination planning. All materials were culturally reviewed before use.

### Ethical considerations

All studies involving human participants were conducted in accordance with the ethical principles of the Declaration of Helsinki. The protocols for each pilot project were approved by the Institutional Review Boards or Ethics Committees at the participating institutions, in accordance with applicable federal regulations and institutional policies. Written informed consent was obtained from all participants prior to study enrollment. Data confidentiality was maintained through coded identifiers and secure servers.

### Reproducibility and transparency measures

With scientific oversight by the AC, reproducibility and transparency will be fostered through preregistration of protocols and analysis plans, public availability of deidentified datasets after publication, sharing of survey instruments and intervention manuals, reporting of all exclusions and attrition, and independent monitoring of data quality.

## Results

Table 2 provides a summary of the progress of the iCCaRE Consortium by September 2024. By the end of the 2-year award, the consortium had met or exceeded all benchmarks proposed for the consortium. The status of each project is provided next.

**Table 2.** iCCaRE Consortium scholarship and research productivity.

Research scholarship	Benchmarks	2-Year accomplishments
Consortium-supported research projects	5	2
Consortium additional pilot projects	2	9
URM investigators	15	26
Presentations at national/international conferences	25	47
Peer-reviewed publications	8	11
Grant submissions	5	27
Grants awarded	3	12
Community reports	2	2
Community presentations	6	66

NOTE: Summary of the iCCaRE Consortium’s research productivity and capacity-building achievements during the initial 2-year funding period. Metrics include consortium-supported projects, pilot studies, underrepresented minority (URM) investigators, scientific presentations, peer-reviewed publications, grant submissions and awards, community reports, and community engagement activities. The table demonstrates the consortium’s contributions to collaborative research infrastructure, translational scholarship, and community-engaged prostate cancer disparities research.

### Project 1: The point of prostate cancer diagnosis Virtual Realty Assistant for Black Men conducted at Mayo Clinic

The iCCaRE project 1 focused on developing an integrated AI and virtual reality (VR) technology tool, Virtual Realty Assistant (ViRA), to provide SDOH navigation, psycho-oncology support, and emotional support for Black men at the point of prostate cancer diagnosis (PPCD). ViRA was codesigned with Black prostate cancer survivors and an interdisciplinary team through structured planning, iterative prototyping, and CAB input, with the intervention delivered through an AI predictive analytic and recommender system. The *emotional support intervention* provides an empathic rapport with prostate cancer survivors through self-disclosure and sharing of survivorship stories. The *psycho-oncology support intervention* provides psychoeducation about the PPCD experience, reifies and concretizes the PPCD experience, and fosters hope. Basic tenets of Problem-Solving Therapy (38) were used for the intervention, including maintaining a positive orientation (e.g., “I can get through this”), using a rational problem-solving style, and self-enhancing statements. Notably, Problem-Solving Therapy is an empirically supported treatment for distress in cancer (39–41). The *SDOH screening and navigation tool* was developed to identify participants’ needs and appropriately connect them with relevant support services and resources in their communities. Alpha testing was conducted in Jacksonville, Florida, and included a total of 10 participants. Twenty-two participants completed the beta testing phase to document the efficacy of ViRA, with ten (10) completing the follow-up interviews. Data collection included user engagement metrics, PROs (e.g., distress, understanding of diagnosis), and qualitative feedback. Descriptive statistics summarized feasibility and engagement, whereas pre–post comparisons assessed changes in PROs. Qualitative thematic analysis was conducted on participant feedback. ViRA was successfully implemented across clinical and community settings, demonstrating high feasibility and usability. Participants reported improved understanding of diagnosis, increased emotional support, and enhanced access to SDOH resources. Qualitative findings highlighted improved patient confidence and reduced initial distress at diagnosis. The effectiveness of the final PPCD ViRA is currently being evaluated through pragmatic clinical trials.

### Project 2: Care at home for Black men conducted at Mayo Clinic

The iCCaRE project 2 aimed to evaluate the feasibility, acceptance, and impact of a new model of cancer care, advanced care at home, by exploring patients’ choices relative to the place of prostate cancer treatment, in the infusion unit or at home (aim 1). The project also aimed to establish the acceptance and impact on PROs and health-related quality of life (HRQoL) in Black men with advanced prostate cancer (aim 2). For aim 1, 50 Black male patients with prostate cancer were enrolled from the Mayo Clinic Florida genitourinary oncology practice. Participants completed a structured questionnaire assessing preferences for treatment location (infusion center vs. home), as well as perceived barriers and advantages associated with each care setting. A subset of 10 patients participated in in-depth semistructured interviews to further explore these themes. In addition, a focus group was conducted with five community advocates to incorporate community-informed perspectives. Preliminary results from focus group sessions indicated a preference for receiving cancer therapy in the home setting as much as possible. For Aim 2, 20 patients completed standardized questionnaires at two prespecified time points—following 8 weeks of in-clinic care and again after 8 weeks of home-based care—to assess key study endpoints. These assessments captured patient-preferred treatment location and level of comfort with receiving injections in the home, as well as broader patient-reported experience domains using the Patient Preference Questionnaire. The perceived value of care was evaluated using the “Was It Worth It” instrument, whereas functional status was assessed with the EORTC QLQ-F17. Symptom burden and treatment-related toxicities were measured using the PRO-CTCAE, and the impact of side effects on patients’ daily lives was captured using the GP5 item. In parallel, clinical outcomes were collected over the same intervals, including the proportion of patients experiencing at least one grade ≥3 adverse event, as well as healthcare utilization metrics such as emergency department visits and hospitalizations. A total of 18 participants were included in the analysis (Arm A: at-home care, *n* = 10; Arm B: in-clinic care, *n* = 8). PROs demonstrated consistently high ratings for communication, convenience, emotional support, and overall care experience across all follow-up periods, with no safety signals observed, including no cancer-related hospitalizations or emergency room visits. The next phase of this project is a pragmatic, single-arm clinical trial to test a care delivery model where Black patients with advanced or metastatic prostate cancer will be offered the opportunity to receive part of their treatment within their home environment.

### Project 3: Survivorship care plan ViRA for Black men conducted at City of Hope

The iCCaRE project 3 focused on developing and implementing a tailored Survivorship Care Plan (SCP) for Black men. A mixed-methods approach was used to develop a prostate cancer SCP tailored to ethnically diverse Black men using clinicians’ key informant interviews and a survey of prostate cancer Black survivors. The SCP was first informed by the literature (42) and the ASCO template (canceradvocacy.org), with strong input and guidance from iCCaRE CAB members, who are prostate cancer survivors. A mixed-methods feasibility study included formative qualitative interviews and quantitative assessments using validated PRO measures (FACT-G, EQ-5D, and SDOH Distress Scale). A total of 139 participants were enrolled. Descriptive statistics summarized baseline characteristics and HRQOL scores, whereas pre–post analyses assessed changes in HRQOL domains. Subgroup analyses were conducted among participants with lower baseline HRQOL (≤25th percentile). At baseline, participants reported generally high HRQOL with low impairment across EQ-5D domains; modest improvements were observed after the intervention. FACT-G analyses demonstrated trends toward improved physical, social, emotional, and functional well-being. Subgroup analyses revealed clinically meaningful improvements among participants with lower baseline HRQOL, with the greatest gains observed in social and physical well-being. Variability in SDOH-related stressors was observed, particularly in healthcare access and cost. Additional analyses are ongoing.

### Project 4: Social determinants of migrant health for Black immigrants conducted at Georgia College

**Figure 4.**
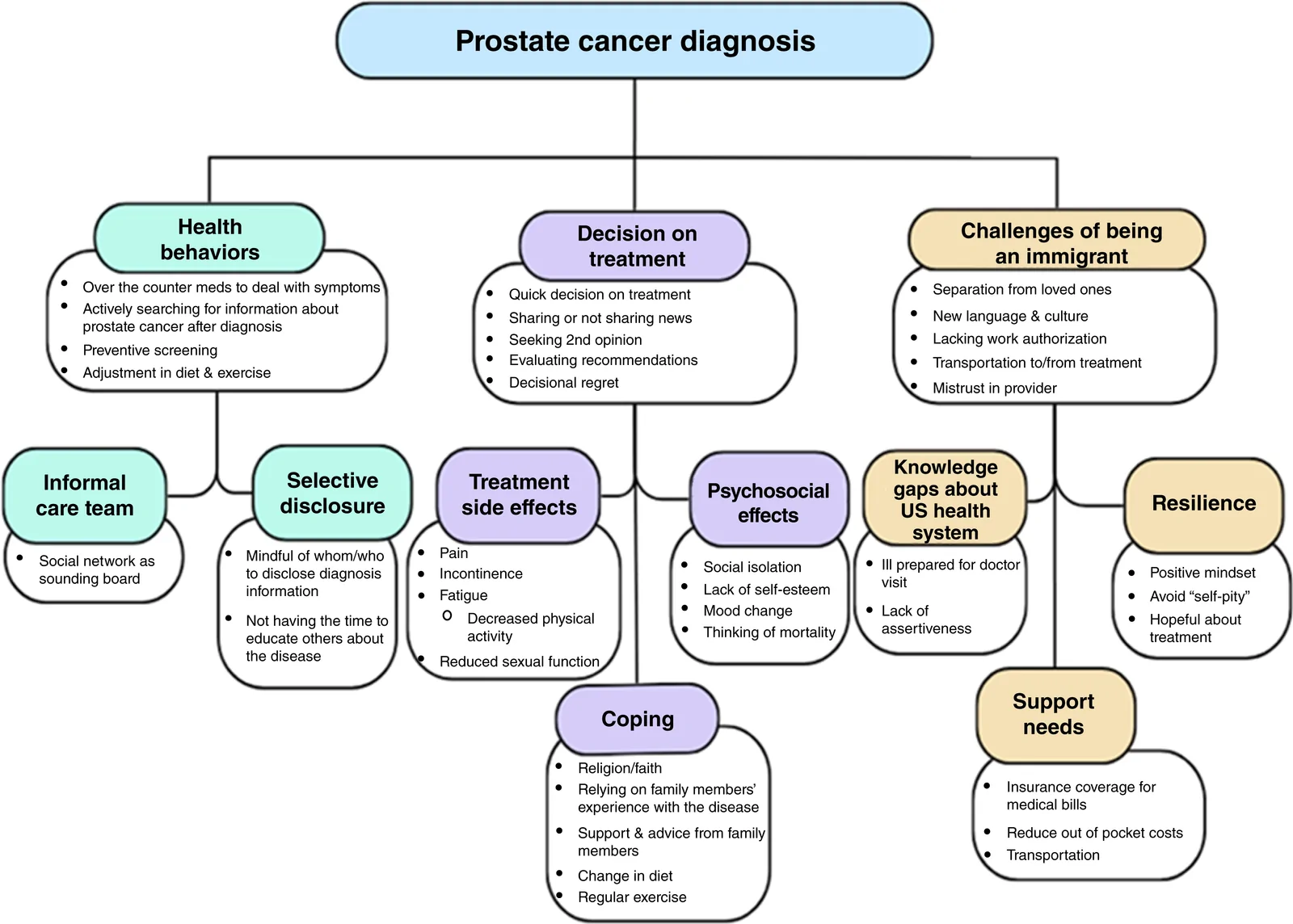
Emerging theoretical model of social determinants of migrant health (project 4). Grounded theory–derived model illustrating the interplay of social, cultural, and structural determinants influencing prostate cancer care and survivorship among SSA immigrant men. The model highlights key domains including health-seeking behaviors, decision-making processes, psychosocial coping (e.g., faith and family support), and structural barriers such as healthcare access and cultural navigation, demonstrating their collective impact on survivorship outcomes.

The iCCaRE project 4 focused on examining the health-seeking behaviors, decision-making about care and treatment, psychosocial effects, and coping mechanisms among prostate cancer survivors who are SSA immigrants. A qualitative grounded theory study was conducted using in-depth, semistructured interviews with prostate cancer survivors. Data were transcribed and analyzed using iterative coding and constant comparative methods. Thematic analysis was used to identify key psychosocial, cultural, and structural factors influencing survivorship. Findings revealed that survivorship experiences were shaped by a strong reliance on faith, family support systems, and cultural beliefs. Participants often made rapid treatment decisions but reported varying degrees of decisional regret. Structural barriers, including healthcare system navigation challenges, cultural differences, and access to care, significantly influenced outcomes. An emerging theoretical model highlighted the interaction between social, cultural, and health system factors (see Fig. 4). This study has been expanded to identify sociobehavioral factors affecting care and treatment among Caribbean immigrant men and to design tailored interventions to reduce health disparities experienced by Black immigrants in the United States.

### Project 5: Assessment of biological determinants of psychoneurologic symptom clusters conducted at Covenant University in Nigeria

**Figure 5.**
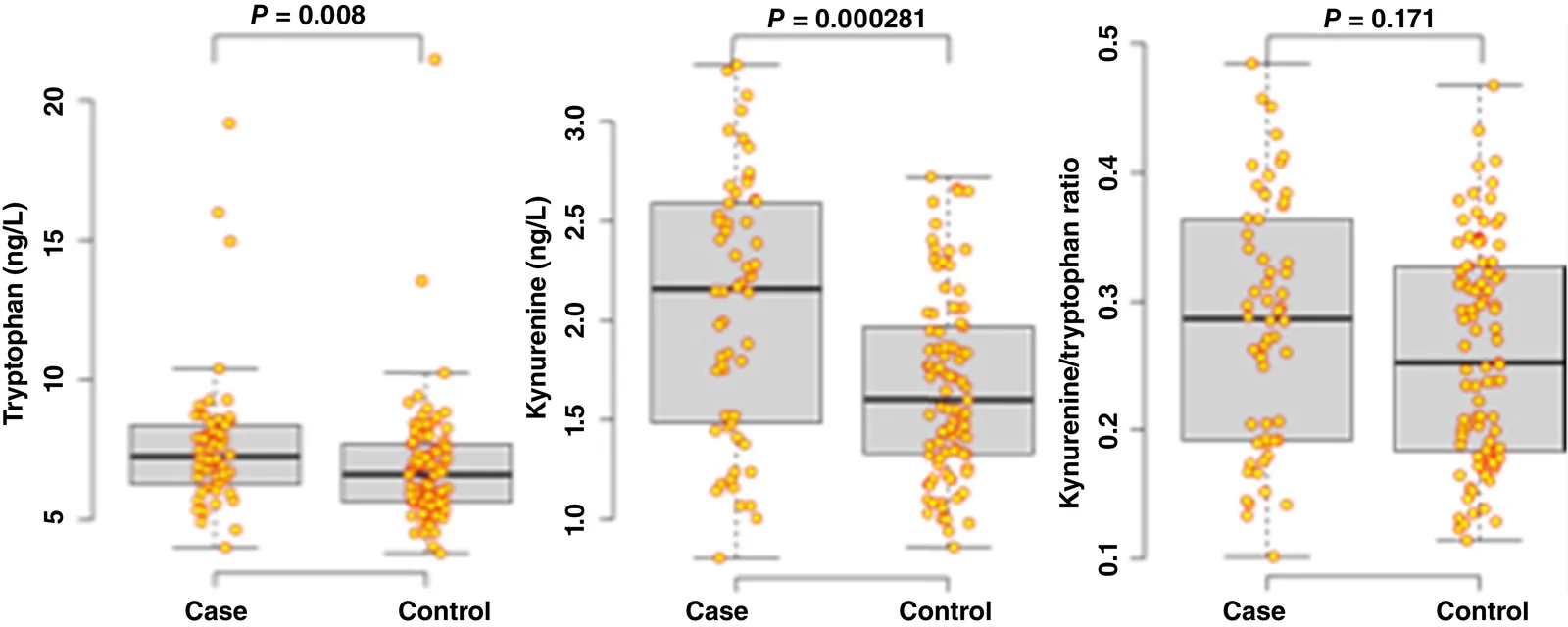
Tryptophan–kynurenine pathway biomarkers in prostate cancer survivors and controls (project 5). Comparison of circulating levels of tryptophan, kynurenine, and the kynurenine/tryptophan ratio between prostate cancer survivors and age-matched controls. The figure demonstrates elevated kynurenine pathway activity among survivors, suggesting altered immune regulation and stress-related metabolic processes that may contribute to symptom burden and survivorship outcomes.

The goal of iCCaRE project 5 was to understand the intersectionality between biology and psychosocial drivers of prostate cancer outcomes among ethnically diverse Black men globally. Controls were age-matched men without prostate cancer and PSA <4 ng/mL. A total of 250 prostate cancer survivors from Nigeria, Benin Republic, Ghana, Niger Republic, Ivory Coast, and Liberia participated in this study. Biospecimens were analyzed for tryptophan, kynurenine, and related metabolites using standardized laboratory assays. Descriptive statistics summarized biomarker distributions, and group comparisons were conducted using inferential statistical tests. Correlation analyses explored relationships between biomarkers and symptom burden. Prostate cancer survivors exhibited elevated kynurenine pathway activity compared with controls, indicating increased immune modulation and stress-related metabolic changes (Fig. 5). This ratio indicates that there is a diversion of tryptophan away from serotonin toward the kynurenine pathway, thereby promoting immune tolerance by inhibiting T-cell proliferation and inducing regulatory T cells (Treg). This allows tumors to evade immune detection and destruction (43). In addition, kynurenine metabolites like quinolinic acid are neurotoxic and have been implicated in psychologic conditions like major depressive disorder (44). Importantly, the acceleration of the conversion of tryptophan to kynurenine is stimulated by stress (45). Such biomarkers, like kynurenine/tryptophan, offer the potential for developing precision interventions to improve the quality of life among prostate cancer survivors. This study has been expanded to investigate the genometabolomics landscape of prostate cancer survivorship among ethnically diverse Black men.

These results provide early signals of intervention feasibility and patient-centered impact. Findings from the iCCaRE Consortium will be disseminated through multiple channels, including peer-reviewed publications, presentations at national and international scientific conferences, and community-focused dissemination through established partnerships with CBOs. Community dissemination strategies include stakeholder briefings, culturally tailored educational materials, and engagement events to ensure findings are accessible and actionable for affected populations.

## Discussion

A significant number of Black men undergo the CaPCaS journey globally, from diagnosis to treatment, survivorship, and unfortunately, end-of-life phases. Supporting Black men in these crucial phases requires a patient-centered, community-centric, and comprehensive approach. Through the iCCaRE Consortium, we brought together all prostate cancer stakeholders to effectively address prostate cancer disparities globally. The Consortium focused on reducing disparities associated with prostate cancer diagnosis, treatment, and survivorship, and improving PROs related to prostate cancer. Within 2 years, the Consortium facilitated the design of five broad-based, translational studies to address prostate cancer disparities experienced by Black men based on the SOS. We successfully leveraged our unique resources, expertise, and diverse stakeholders globally to fill the knowledge gap on the SDOH, psychosocial, and biological factors affecting CaPCaS among men of African ancestry, including documenting if there are ethnic differences among the subpopulations of Black men.

Within 2 years, the iCCaRE projects and Consortium as a whole addressed critical barriers affecting prostate cancer disparities. At the health care system level, all the projects addressed lack of access, technology gaps, organizational health literacy, and lack of cultural sensitivity. At the provider level, project 2 focused on care at home by a culturally responsive team, which has addressed provider bias (including discrimination and marginalization of Black men) and cultural humility. At the individual level, Projects 1, 2, 3, and 4 addressed SDOH barriers, health literacy, fear, mistrust, and cultural and/or language barriers. Project 5 focused on biological factors by addressing the underrepresentation of genetic information of Black men as well as the limited inclusion of genomic data from SSA countries for precision/personalized medicine. The iCCaRE Consortium also addressed critical barriers affecting the QOL of Black men affected by prostate cancer. For example, Project 2 addressed the limited multidisciplinary oncology practice accessible to Black men through the novel care at home project. Projects 2 and 3 addressed the lack of surveillance, monitoring, or follow-up often experienced by Black men. All the iCCaRE projects addressed the lack of patient-centered models to guide the improvement of QOL, as well as the lack of PRO measures tailored to Black men.

Another novel component of the iCCaRE Consortium is the use of AI intervention programs that are intelligent, discreet, accessible, connected, and personalized to support Black prostate cancer patients. In contrast to standard clinic-based approaches, which often provide limited, nontailored psychosocial support at the PPCD and during survivorship, ViRA delivers culturally relevant, immersive, and personalized support designed to address emotional, informational, and social needs in real time. In the digital age of healthcare, greater efficiencies and improved health outcomes can be accelerated by advanced data analytics and immersive visualization powered by AI. This acceleration is predicated on the synergy of clinical and data science expertise, innovative use of state-of-the-art AI, VR/augmented reality (AR) technologies, and efficient use of patient, provider, institutional, and health system factors. The consortium capitalized on the breadth and depth of these factors to develop a number of intelligent, pervasive iCCaRE ViRAs that aim to help Black men from the PPCD through survivorship. ViRA is a computer-generated, AI-enabled virtual assistant accessible through smartphones/tablets and VR/AR headsets. Each iCCaRE ViRA (PPCD/prostate cancer SCP) is personalized for Black men to help them make more informed decisions about their care and survivorship based on their data and personal preferences. The ongoing ViRA pragmatic clinical trial will establish direct quantitative comparisons of clinical outcomes between ViRA and standard of care, further evaluating its effectiveness in improving patient-centered outcomes across the prostate cancer continuum.

The findings from this study primarily reflect process outcomes and early signals of effectiveness rather than definitive clinical impact. Although preliminary results suggest improvements in patient experience and feasibility of interventions, conclusions about the reduction in disparities require confirmation through ongoing trials and longitudinal analyses. Several limitations should be considered. The multisite nature of the consortium introduced coordination challenges and variability in implementation across settings. Cross-national regulatory requirements added complexity to study execution. Additionally, the heterogeneity of study populations and modest sample sizes in feasibility studies may limit generalizability. These factors are inherent to early-phase, multilevel intervention research and will be addressed in future larger-scale studies. Ongoing efforts will expand cohort size, rigorously evaluate clinical outcomes, and accelerate dissemination across both academic and community settings to advance health equity.

### Conclusion

Although several federally funded prostate cancer consortia have advanced understanding of the biological and genetic underpinnings of disease, critical gaps remain in addressing survivorship, patient-centered outcomes, and the multilevel factors driving disparities. The iCCaRE Consortium was established to address these unmet needs through an SOS framework that integrates clinical, social, behavioral, and biological determinants of care. By bringing together survivors, community leaders, clinicians, and transdisciplinary scientists, iCCaRE advances a collaborative, translational, and globally informed approach to improving the prostate cancer care continuum, from diagnosis through survivorship. The interventions developed through this consortium are designed to enhance patient support, inform decision-making, and improve quality of life in clinical and community settings. As these efforts mature, they have the potential to contribute to improved outcomes and to inform scalable strategies aimed at reducing prostate cancer disparities among Black men.

## Supplementary Material

Supplementary Data iCCaRE Consortium membership list

## Data Availability

The deidentified data generated and/or analyzed during this study are available from the corresponding author upon reasonable request and subject to Institutional Review Board approval, data use agreements, and applicable participant confidentiality protections. Because the study includes multisite human participant data collected across the United States and SSA, some restrictions may apply to data sharing in accordance with local regulatory and ethical requirements.
