# Supplementary material for: Advancing Prostate Cancer Survivorship through Transatlantic Consortium Science: The iCCaRE Initiative
Source: Cancer Res Commun. 2026 Sep 3;6(9):2067–78. doi: 10.1158/2767-9764.CRC-26-0165 (PMC13539179; doi:10.1158/2767-9764.CRC-26-0165)
Supplement: Supplementary file 1 [file crc-26-0165_supplementary_data_suppds.docx]

**Name: Folakemi Odedina, PhD**  
Project Role: MPI

Research Identifier: 0000-0003-3796-1385 (ORCID)

Contribution to project: Dr. Odedina provides overall scientific direction and administrative leadership for the award. She is also the Full Project 1 (FP1) MPI and overall Consortium Director

**Name: Che Ngufor, PhD**  
Project Role: MPI

Research Identifier: 0000-0001-5935-5744 (ORCID)

Contribution to project: Dr. Ngufor is DHHS co-lead, iCCaRE Co-MPI on Full Project 1 and Associate Professor of Biomedical Informatics at Mayo Clinic. Dr. Ngufor leads the overall DHHS activities and Full Project 1 efforts regarding ViRA and AI integration components.

**Name: Christopher Williams, MD**  
Project Role: iCCaRE Consortium Medical Director

Contribution to project: Dr. Williams provides overall medical guidance and oversight of the FP1 project. He provides his clinic for recruitment and serves as the iCCaRE Consortium Medical Director. 
 
**Name: Mary Ellen Young, PhD**

Project Role: Consultant

Research Identifier: N/A (ORCID)

Contribution to project: Dr. Young is the FP1 MaMS representative She also assist in FP3 by ensuring the methodology is applied in a robust manner and provides feedback on study instruments, coding, emerging themes and interpretive framework.

**Name: Floyd Willis, MD**  
Project Role: iCCaRE Consortium Co-Investigator  
Research Identifier: N/A (ORCID)

Contribution to project: Dr. Willis is a family medicine and community physician who provides resources to assist with medical chart review of eligible patients and as the PES representative, assists with outreach activities.   
 
**Name: Adam Kase, MD**

Project Role: iCCaRE Consortium Co-Investigator

Research Identifier: 0000-0002-1862-8467 (ORCID)

Contribution to project: Dr. Kase is a physician scientist and provides clinical feedback for FP1.

**Name: Arnold J. Merriweather**

Project Role: Community MPI

Research Identifier: N/A

Contribution to project: Mr. Merriweather is the leader of the community advisor, provides input on Full Project 1, the administrative core (AC), and in the partnership and engagement core (PES).

**Name: Emelina Asto-Flores, MS  (no longer affiliated)**

Project Role: iCCaRE Consortium Manager

Research Identifier: 0009-0008-0942-8755

Contribution to the Project: Manages the day-to-day activities of the consortium and keeps all MPIs abreast of changes and ensures the progress of the consortium.

**Name: Dee Glaser-Boivin, RN**

Project role: Clinical Research Coordinator

Research Identifier: N/A

Contribution to the project: Ms. Glaser-Boivin coordinates the clinical aspects of FP1 and FP2 projects.

 Ne
**Name: Jada K. Melton, MPH**

Project role: Program Coordinator

Research Identifier: N/A

Contribution to the project: Ms. Melton helps coordinate the logistical aspects of the project. She works closely with the project’s Clinical Research Coordinator.

**Name: Quincy Wimberly, MSHI**

Project role: Clinical Research Coordinator

Research Identifier: N/A

Contribution to the project: Mr. Wimberly helps with uploading the ViRA application to the Meta Quest headsets and ViRA troubleshooting in addition to outreach efforts at the participating American Legion Posts.

**Name: Michelle Fudge**  
Project Role: CHERP Program Manager  
Research Identifier: N/A (ORCID)

Contribution to project: Mrs. Fudge provides operational oversight over the iCCaRE Consortium.

**Name: Roxana Dronca**

Project Role: Principal Investigator (PI)

Researcher Identifier: 0000-0002-5345-2268 (ORCID)

Nearest person month worked: 0.72

Contribution to Project: Dr. Dronca leads and provides oversight for full project 2, including the development of

the clinical trial protocol, leading the CCBW program, training and overseeing the clinical research team.

**Name: Trevanne Matthews Hew, MD**

Project Role: Co-Investigator (CI)

Researcher Identifier: 0000-0002-9085-9085-5568 (ORCID)

Contribution to Project: Dr. Matthews Hew is a member of Full Project 2 and a member of the Consortium Administrative Core. Dr. Matthews-Hew has assisted with protocol writing, questionnaire development, participant study identification, and patient selection tools.

**Name: Former Senator Antony Hill**

Project Role: Community Multiple Principal Investigator (MPI) Researcher Identifier: NA

Contribution to Project: Former Senator Hill is the community MPI on Full Project 2.

**Name: Michael Maniaci, MD**

Project Role: Co-Investigator (CI) Researcher Identifier: NA

Contribution to Project: Dr. Maniaci is the Director of the Mayo Clinic Center for Digital Health and the Mayo Clinic Florida (MCF) Director of the Advanced Care at Home Program (ACH) and a member of Full Project 2 and directs the Advanced Care at Home (ACH) Program.

**Name: Adam Kase, MD**

Project Role: Trainee/Co-Investigator (CI) Researcher Identifier: NA

Contribution to Project: Dr. Kase is a member of Full Project 1 and Full Project 2 and has assisted with protocol writing, questionnaire development, patient selection tools, and participant study identification.

**Name: Tracie Scales, MS**

Project Role: Senior Research Protocol Specialist

Researcher Identifier: NA

Contribution to Project: Ms. Tracie coordinates the overall management of the clinical trial development and activation process for FP2 and FP5

**Name: Lucia Nolan, Pharm.D., R.Ph.**

Project role: Pharmacy Coordinator

Research identifier: NA

Contribution to the project: Ms. Lucia identifies drug therapy for safe home injection/infusion administration

while organizing the medication delivery process for the home administration phase.

**Name: Kaila Falzon**

Project role: Senior Program Coordinator

Research Research identifier: NA

Contribution to the project: Ms. Kaila coordinates the regulatory aspects of the project.

**Name: Dr. Ernie Kaninjing**

Project Role: PI

Research identifier: 0000-0003-4250-9756

Contribution to the project: Dr. Kaninjing determines the overall direction of the study including protocol development, training of staff, and working with cancer registries to seek protocol approval for the study. Completed data request applications with Florida Cancer System, Florida Department of Health and Texas Cancer Registry.

**Name: Dr. Gladys Asiedu**

Project Role: CO PI

Research Identifier: ORCID ID: 0000-0002-2710-5203

Contribution to the project: Dr. Asiedu contributed in Protocol development, completing IRB material, seeking approval from Mayo Clinic IRB.

**Name: Mr. Patrick Beckford**

Project Role: Community PI

Research identifier: NA

Contribution to the project: As a consumer advocate originally from the Caribbean Island of Jamaica, Mr. Beckford provides input on interview guide and development of study materials. He also participates in monthly training and acts as first level consumer protector.

**Name: Mr. Emmanuel Agboola**

Project Role: Community PI

Contribution to project: Mr. Agboola is a consumer advocate originally from sub-Sahara Africa. He provides inputs to the development of study materials like the interview guide and serves as the first level 	consumer protector. He also participates in monthly team meeting.

**Name: Kaitlin Van Voorhis**

Project Role: Graduate Student/Research Assistant

Contribution to Project: Works as research assistant ensuring protocols are up to date and collaborates with other iCCaRE Research Assistant to ensure constant communication and integration of iCCaRE activities. She developed the recruitment Flier requested by Texas Cancer Registry as part of the data request application.

**Name: Mr. Daniel Lee**

Project Role: Co Investigator

Contribution to project: Participated in the data request application for the Texas Cancer Registry and responded to inquiries for details about data analysis plan.

**Name: Rakenya Hinsom**

Project Role: ESI

Research Identifier:

Contribution to the project: Descriptive data analysis of dataset from cancer registry; contribution to qualitative data analysis and interpretive framework.

**Name: Prof. Solomon O. Rotimi (PhD.)**

Project Role: Principal Investigator/Team lead

Researcher Identifier (e.g. ORCID ID): 0000-0002-3678-9977

Contribution to Project: Design and provide overall direction of the project, as well as supervision of the project. Prof. Rotimi is also the Full Project 4 (FP4) MPI.

**Name: Prof. Usman M. Aliyu (PhD.)**

Project Role: Collaborator

Researcher Identifier (e.g. ORCID ID):  0009-0006-1452-5782

Nearest person month worked: 1.8

Contribution to Project: Contributing available resources at National Institute for Cancer Research and Treatment, Abuja, Nigeria and recruitment of participants.

**Name: Dr. John A. Copland**

Project Role: Collaborator

Researcher Identifier (e.g. ORCID ID): 0000-0001-5091-6242

Contribution to Project: Provide technical and operational oversight in specific aspect of the projects.

**Name: Capt. Ayinde Yahaya Ayinde (Rtd)**

Project Role: Community investigator

Researcher Identifier (e.g. ORCID ID): NA

Contribution to Project: Community Advocacy and managing community-based initiatives.

**Name: Dr. Clayton Yates, (PhD.)**

Project Role: Collaborator

Researcher Identifier (e.g. ORCID ID):  0000-0001-5420-9852

Contribution to Project: Contributing available equipment and other technical expert in order to achieve the project's objective.

**Name: Prof. Emeka J. Iweala (PhD.)**

Project Role: Collaborator

Researcher Identifier (e.g. ORCID ID): 0000-0002-1393-1416

Contribution to Project: Contributing available equipment at the CApIC-ACE inorder to achieve the project's objective. Recruitment of participants, collection of biospecimen and survey instruments.

**Name: Dr. Opeyemi C. De Campos (PhD.)**

Project Role: Research Coordinator

Researcher Identifier (e.g. ORCID ID): 0000-0001-9094-5670

Contribution to Project: Project coordination.

**Name: Dr. Abimbola F. Onyia (PhD.)**

Project Role: Postdoctoral Fellow

Researcher Identifier (e.g. ORCID ID): 0000-0002-6040-9671

Contribution to Project: Data and laboratory analysis

**Name: Esther Olasehinde (MSc.)**

Project Role: Research Assistant

Researcher Identifier (e.g. ORCID ID): 0009-0004-0439-2766

Contribution to Project: Data and laboratory analysis

**Name: Gerardo Colon-Otero, MD**

Project Role:  Principal Investigator

Researcher Identifier (e.g., ORCID ID):  0000-0002-9159-2421

Contribution to Project: Directed the clinical trial. Participated in every 2 weeks laboratory meetings. Analyzed preliminary data. Directed every other week team meetings with colleagues from Nigeria. Obtained history from Mayo Clinic patient participants and enrolled them in the study. Interpreted laboratory results for the patients.

**Name: Keith Knutsen, PhD**

Project Role:Co-Investigator, Directs laboratory performing immunological studies

Researcher Identifier (e.g., ORCID ID):  N/A

Contribution to Project: Directed the laboratory performance of ELISA assays for detecting levels of antibodies

**Name: Mahesh Gokara, PhD**

Project Role: Post-Doctoral Researcher in Dr. Knutson’s laboratory

Researcher Identifier: 	0000-0001-7095-887X

Contribution to the Project: Performed the laboratory experiments (setup ELISa) in Dr. Knutson’s laboratory.

**Name: Sandra Casanova Leyva M.B.A**

Project Role: Sr. Program Coordinator

Researcher Identifier (e.g., ORCID ID):  0009-0003-8614-3685

Contribution to Project: Enrolled and consented patients for the study. Entered data in RedCap. Organized patients appointments and collected data. Participated in all meetings related to the study. Will train Nigeria personnel of the specifics of data collection, patient consenting and study requirements.

**Name: Alex Cattnell**

Project Role: Associate Clinical Research Coordinator

Researcher Identifier (e.g., ORCID ID):  N/A

Contribution to Project: Enrolled and consented patients for the study. Entered data in RedCap. Organized patients appointments and collected data.

**Name:  Luisa Mitchell**

Project Role: Clinical Research Coordinator

Researcher Identifier (e.g., ORCID ID):  N/A

Contribution to Project: Enrolled and consented patients for the study. Entered data in RedCap. Organized patients appointments and collected data.

**Name: Melissa Doty**

Project Role: Associate Clinical Research Coordinator

Researcher Identifier (e.g., ORCID ID):  N/A

Contribution to Project: Enrolled and consented patients for the study. Entered data in RedCap. Organized patients appointments and collected data.

**Name: Carolyn R. Lindeman  R.N.**

Project Role:  RN Research Navigator

Researcher Identifier (e.g., ORCID ID):  N/A

Contribution to Project: Assist with participant screening.

**Name: Claudia Valles Duarte M.S.**

Project Role: Program Coordinator

Researcher Identifier (e.g., ORCID ID): N/A

Contribution to Project: Assist with participant screening.

**Name: Hanna Sledge**

Project Role: Statistical Programmer

Researcher Identifier (e.g., ORCID ID):  N/A

Contribution to Project: Assist with EDC system modifications and building CRFs.

**Name:  Ademoola Popoola, MD**

Project Role: Co-Principal Investigator, Leads research effort at University of Ilorin, Ilorin, Nigeria

Researcher Identifier (e.g., ORCID ID):  0000-0002-1850-2223

Contribution to Project: Worked on getting University of Ilorin IRB approval, approval of Nigeria government required NAFDAC process, participated in iCCARE meetings.

**Name:  Cassandra Moore, MD**

Project Role:  Co-Investigator, Directs study accrual at Mayo Clinic Arizona

Researcher Identifier (e.g., ORCID ID):  N/A

Nearest person month worked:  0.14

Contribution to Project:  Accrue patients at Mayo Clinic Arizona

**Name:  Kim Barbel Johnson D.O. M.P.H.**

Project Role: Co-Investigator

Researcher Identifier (e.g., ORCID ID):  0000-0002-8982-0958

Contribution to Project: Participated in all study meetings and provided guidance in procedures needs for both studies.

**Name: Trevanne R. Matthews Hew, M.B.B.S**.

Project Role: Co-Investigator

Researcher Identifier (e.g., ORCID ID):  N/A

Contribution to Project: Obtained history from Mayo Clinic patient participants and enrolled them in the study

**Name: Ewan Cobran , PhD**

Project Role: Interim Contact PI

Researcher Identifier: 0000-0002-1842-312X

Nearest person month worked: 0.00

Contribution: Dr. Cobran is currently filling in as interim contact PI for PP6, providing scientific and study oversight of the study in Arizona. Dr. Cobran’s research focuses on eliminating prostate cancer disparities, increasing understanding of prognostic genetics, enhancing communication between patient and care provider, and improving genetic decisional support for men with localized prostate cancer.

**Name: Opeyemi Bolajoko, PhD**

Project Role: Interim Contact PI

Researcher Identifier: 0000-0002-9841-4476

Contribution: Dr. Bolajoko is currently filling in as interim contact, providing scientific guidance and study oversight of the study within the Florida site. Dr. Bolajoko has years of global prostate cancer disparities research and her expertise in cancer disparities is a suiting role to lead this project, as interim PI in Florida.

**Name: Dottington Fullwood, EdD  (No longer involved)**

Project Role: Previous PP6 PI from 09/30/24 – 10/04/2025

Researcher Identifier: 0000-0001-6077-3302

Contribution: Dr. Fullwood focused on a variety of critical tasks, including community outreach, app development, publication efforts, and refining key project documents. These efforts have been instrumental in propelling the project forward and ensuring alignment with its objectives. The time dedicated to these areas demonstrates a strong commitment to both scientific excellence and community engagement, addressing the project’s evolving needs and setting the foundation for the pilot’s success.

**Name: Amanda Caballero**

Project Role: Program Coordinator

Researcher Identifier: 0009-0001-9013-4779

Nearest person month worked: 0.00

Contribution: Ms. Caballero’s is the program coordinator for Dr. Cobran and has years of experience in health disparities research. Currently she works in providing logistical support for the study in Arizona, by helping the team navigate the study setup process with Dr. Cobran listed as the PP6 PI in Arizona.

**Name: Jade Thompson - Reeves**

Project Role: Research Project Assistant

Researcher Identifier: N/A

Nearest person month worked: 0.1

Contribution: Ms. Reeves is the research project assistant for both Dr. Cobran and Dr. Opeyemi Bolajoko and has community-based participatory research experience. Due to her experience in this field, she will be mainly assisting the team with recruitment support in Florida by engaging with community partners and attending different community outreach events.

**Name: Charles F. Waldon**

Project Role: Consultant

Researcher Identifier: N/A

Contribution: Community scientist. Mr. Waldon, plays a pivotal role in advancing the project, continuing his active engagement in both community outreach and app development. His close collaboration with the PI and survivors provided invaluable feedback on the app’s user experience, ensuring it continues to meet the needs of participants. Mr. Waldon’s efforts have also been crucial in expanding the project’s reach, actively recruiting new survivors and identifying key events and counties for potential recruitment. His unwavering commitment to refining the project’s design and engagement strategies has been essential to the project’s success, making him an indispensable part of the team.

**Name: Kimlin Tam Ashing, PhD Project Role: ReAT MPI**

Researcher Identifier: 0000-0002-6209-508X Nearest person month worked: 1

Contribution to Project: Dr. Ashing is the MPI liaison for the REaT core

**Name: Vinessa Gordon, MPH  (no longer involved)**

Project role: Impact Evaluator Research identifier: NA

Contribution to project: Provides evaluation on all iCCaRE Consortium activities

**Name: Collisa Mahin, MPH.**

Project role: Project Coordinator

Research identifier: NA

Contribution to Project: Collisa supports all the consortium’s CORES and services.

**Name: Pastor John McCall, LCSW**

Project Role: CAB member

Contribution to the Project: Is a CAB member. Provides input on intervention guides on the full projects with a community/ prostate cancer survivor lens

**Name: Dr. Ebenezer Erefah**

Project Role: Community MPI FP3

Contribution to the project: As the consumer advocate, Dr. Erefah provides input on full project #3, including study instruments

**Name: Kathleen Yost, PhD**

Project Role: MaMS co-lead

Research Identifier: 0000-0002-9641-5627

Contribution to the Project: Dr. Yost leads in the MaMS core efforts, she co-leads in reviewing all the documents submitted by the cores and in the collaborative partnership with the DaMAS core.

**Name: Dr. Catherine Oladoyinbo**

Project Role: MaMS Global co-lead

 Research Identifier:N/A

Contribution to the Project: Dr. Oladoyinbo co- leads in the MaMS core efforts, she co-leads in reviewing all the documents submitted by the cores and in the collaborative partnership with the DaMAS core.

**Name: Rickey Carter, PhD**

Project Role: DaMAS Co-lead

Researcher Identifier: 0000-0002-0818-273X

Contribution to project: Dr. Carter is the lead for DaMAS and ensures the data management and statistical analysis needs of the iCCaRE projects are met. He also collaborates with MaMS to ensure synergy across iCCaRE Consortium.

**Name: Getachew Dagne, PhD**

Project Role: DaMAS Co-lead

Researcher Identifier: N/A

Nearest person month worked: N/A

Contribution to project: Dr. Dagne is the co-lead for DaMAS and ensures quantitative study instruments are robust and the statistical needs of the cores are met.

**Name: Jordan P. Reynolds MD,**

Project Role: BiRC Co-lead

Researcher Identifier: 0000-0001-7455-6122

Contribution to Project: Procurement, storage management, and distribution of biospecimens for the Consortium and non-consortium investigators.

**Name: Kayode Adeniji, MBBS**

Project Ro/le: BiRC co-Lead

Researcher Identifier: N/A

Nearest person month worked: 0.9

Contribution to Project: Procurement, storage management, and distribution of biospecimens for the Consortium and non-consortium investigators.

**Name: Floyd Willis, MD**

Project Role: PES Co-lead

Researcher Identifier: N/A

Contribution to Project: Oversight of PES core development, community engagement and document implementation

**Name: Renaldo Blocker, Ph.D.**

Project Role: DHCC core member

Research Identifier: NA

Contribution to the Project: Cognitive Engineering expertise

**Name: Timethia Bonner, PhD**

Project Role: DHCC core member

Research Identifier (e.g. ORCID): 0000-0001-9273- 4092

Contribution to the Project: Dr. Bonner has performed work in qualitative coding, analysis, and research Dissemination.

**Name: Kim Walsh-Childers, PhD**

Project role: DHHS member/ consultant

Research identifier: 0000-0002-0050-3359

Nearest person month worked: consultant

Contribution to the project: Dr. Walsh-Childers is a Professor Journalism at the University of Florida. Dr. Walsh Childers review and provide critical methodological advice on DHHS activities.

**Name: Abimbola Odedina**

Project Role: Consultant Research Identifier: N/A

Nearest person Month worked: NA Consultant

Contribution to the Project: Serves as the iCCaRE Consortium Media Director. Provides and coordinates media support and services within the consortium.

**Name: Dr. Damian Francis, PhD**

Project Role: Evaluation Core

Researcher Identifier: NA

Nearest person month worked: 3

Contribution to Project: Dr. Francis leads to planning and evaluation core

**Name: Ivey Breland**

Project Role: Graduate student Researcher Identifier: NA Nearest person month worked: 3

Contribution to Project: Mrs. Breland serves on the planning and evaluation core to monitor the surveys and provide training for consortium.

**Name: Paul Badejo**

Project Role: Clinical Trails Coordinator in Nigeria

Researcher Identifier: N/A

Contribution to Project: Organizes and prepares documents to be submitted to NAFDAC. Mr. Badejo will recruit patients in Nigeria.

**Name: Oluwaseyi Toye**

Project Role: Global Consortium Manager

Research Identifier: N/A

Contribution to the project: Oversees iCCaRE day-to-day activities in Africa. Manages all global coordinators in Africa, as well as global activities and events.

**Name: Jeffery Gainer, MPH, CCRC**

Project role: Consortium Manager

Research Identifier: 0009-0003-5507-0276

Contribution to the project: Jeffery Gainer manages the day to day activities of the consortium and keeps all MPIs abreast of changes and ensures the progress of the consortium.

**Name: Uju Aliche, PhD**

Project role: Early Stage Investigator

Contribution: Provides oversight for cores and projects

Name: Tingyu Zou, PhD

Project Role: Early Stage Investigator

Contribution: Contributes to the consortium
